# High Bandwidth Control of a Piezo-Actuated Nanopositioning System Based on a Discrete-Time High-Order Dual-Loop Framework

**DOI:** 10.3390/s25185754

**Published:** 2025-09-16

**Authors:** Longhuan Yu, Xianmin Zhang, Sergej Fatikow

**Affiliations:** 1 Guangdong Key Laboratory of Precision Equipment and Manufacturing Technology, School of Mechanical and Automotive Engineering, South China University of Technology, Guangzhou 510000, China; hans_longhuan@163.com; 2 Division of Microrobotics and Control Engineering, Department of Computing Science, University of Oldenburg, 26129 Oldenburg, Germany; sergej.fatikow@uni-oldenburg.de

**Keywords:** dual-loop control, piezo-actuated nanopositioning system, discrete-time, state feedback, linear quadratic regulator

## Abstract

Dual-loop control enhances the bandwidth of piezo-actuated nanopositioning systems via inner-loop state feedback controller suppressing lightly damped resonance and outer-loop tracking controller eliminating hysteresis nonlinearity. However, the traditional framework based on the continuous-time low-order model suffers from control performance degradation. To address this issue, this paper proposes a dual-loop control framework based on the discrete-time high-order model. In this framework, the discrete-time linear quadratic regulator extends theoretical bandwidth through simultaneous parameter optimization, and direct discrete implementation of the high-order state feedback controller and an integrator improves control precision by reducing model mismatch and controller discretization errors. Experiments are conducted on a custom-designed piezo-actuated system. Experimental frequency response of the system with the developed framework agrees well with the theoretical one, and the actual bandwidth is improved to 8248 Hz, which is better than 3920 Hz (continuous-time high-order model) and 6610 Hz (discrete-time low-order model), and exceeds open-loop resonant frequency 6352 Hz. Step response and trajectory tracking tests also demonstrate the effectiveness of the developed framework.

## 1. Introduction

Piezo-actuated nanopositioning systems generally consist of the power amplifier, the piezoelectric actuator, the compliant positioning stage, the displacement sensor, and the digital acquisition and control unit. Due to the advantages of nanoscale resolution, high natural frequency, and absence of friction, they have been widely applied in the ultra-precision equipment, such as atomic force microscopes (AFMs) [1], micro-grippers [2], and fast steering mirrors [3]. However, their actually achievable control bandwidth is greatly hindered by the lightly damped resonance and hysteresis nonlinearity.

To overcome these limitations, inverse model-based feedforward control is the most straightforward and effective method [4,5,6]. However, modeling uncertainties, external disturbances, and inverse calculation errors make it hard for them to obtain a satisfactory result. Although combining the above feedforward control methods with a feedback controller [7,8,9] can achieve high-bandwidth control with enhanced robustness and disturbance rejection, precise modeling of complex hysteresis phenomena remains a significant challenge [10].

To avoid the hysteresis modeling, the dual-loop feedback control framework [11,12] is popularly adopted for the piezo-actuated nanopositioning system to ensure a broad bandwidth and achieve high-precision tracking performance. In this architecture, the inner-loop utilizes active damping controllers, such as positive position feedback (PPF) [13], positive velocity and position feedback (PVPF) [14], integral resonant control (IRC) [15], positive acceleration, velocity, position feedback (PAVPF) [16], and observer-based state feedback [17], to sufficiently suppress the resonance mode. Then, the outer loop utilizes high-gain feedback controllers, such as integral [18], proportional-derivative [19], and proportional-integral [20], to reject disturbances mainly caused by the hysteresis.

Generally, inner-loop damping controllers are designed based on a second-order or third-order model, and then applied to practical systems [17,21]. However, with the consideration of the dynamics of the piezoelectric amplifier and the sensor, as well as the time delay induced by the hardware/software system, the nanopositioning system should be described by a high-order model, rather than a low-order model [22,23]. This model mismatch will cause unsatisfactory control performance [17]. Moreover, the aforementioned dual-loop control schemes rely on continuous-domain design followed by discretization. However, the discretization error cannot be ignored when the sample time is not sufficiently small [24,25,26]. To maximize the bandwidth of closed-loop systems, simultaneous design methods for tuning inner and outer loop controller parameters, such as the analytical approach [27], experimental parameter optimization [28], and the intelligent optimization algorithm [29], are commonly employed. However, these methods exhibit computational inefficiency and fail to obtain optimal control parameters when addressing the high-order controller design problem.

Therefore, to enhance practical control bandwidth for nanopositioning systems with high-order dynamics and hysteresis nonlinearity through the dual-loop control framework, we design a high-order state feedback damping controller based on a high-order model rather than a low-order model to control the practical high-order system. And both the inner-loop damping controller and the outer-loop integral tracking controller are designed directly based on the discrete-time model, rather than being developed through continuous-time modeling followed by discretization. Furthermore, we implement a discrete-time linear quadratic regulator (DLQR) for optimal parameter tuning of a discrete-time high-order controller.

The main contributions are threefold. (1) A discrete-time high-order dual-loop control framework is developed to eliminate low-order modeling and controller discretization errors. (2) The DLQR technique is utilized to flexibly tune the control parameter, enabling the theoretical bandwidth to exceed the first resonant frequency of the open-loop system. (3) The developed control framework is experimentally validated, successfully achieving the theoretically designed high-bandwidth performance with highly consistent amplitude response.

The rest of the article is organized as follows. The considered problem is defined by describing the conventional dual-loop framework in Section 2. Section 3 presents the developed dual-loop framework in detail. Experimental setup, system identification, and experimental results are summarized in Section 4. Finally, conclusions are drawn in Section 5.

## 2. Problem Formulation

### 2.1. Conventional Dual-Loop Control Framework

**Figure 1:**
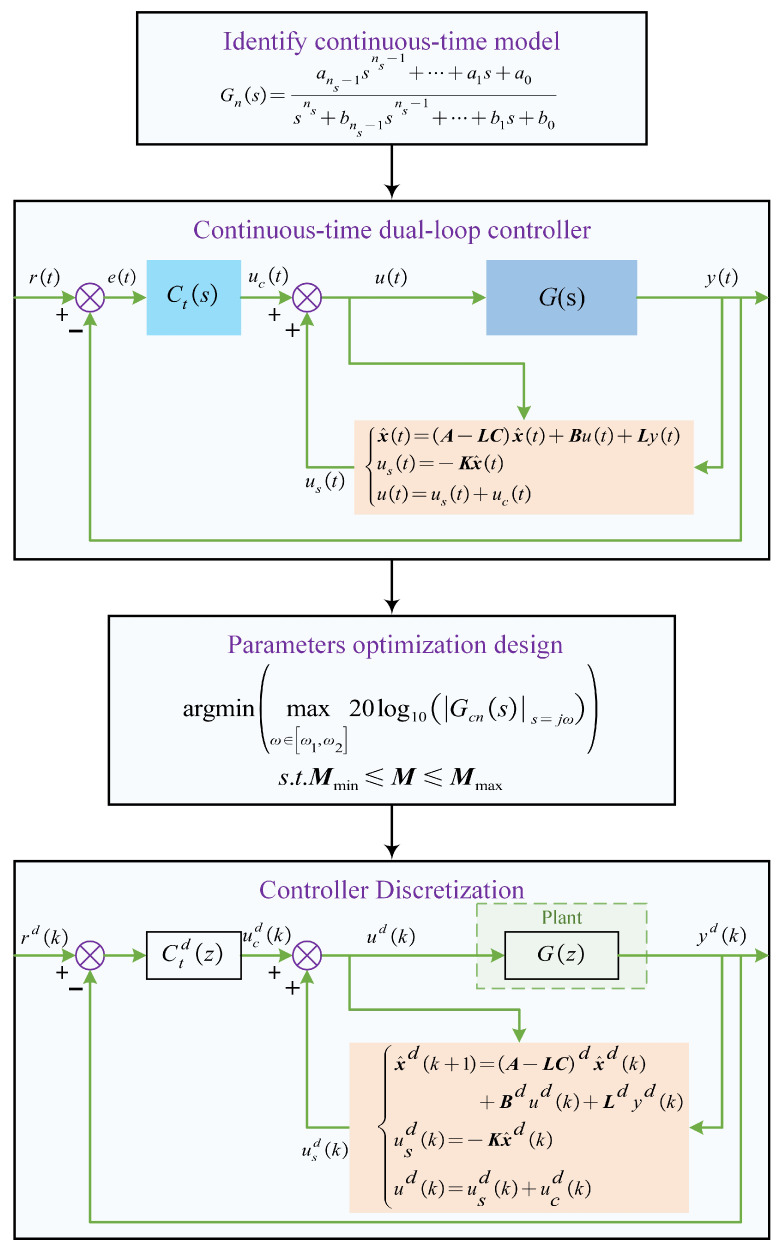
Design procedure of the conventional continuous-time low-order dual-loop control framework.

According to references [12,15,16,17,28], damping and tracking controllers can take various forms and structures. However, their essential function is to modify the closed-loop pole/zero locations to achieve the desired frequency response characteristics. Referring to Tao’s work [17], the design procedure for the conventional dual-loop control framework illustrated in Figure 1 is summarized as follows.

First, from the input voltage u(t) (V) to the output displacement y(t) (μm), the open-loop system model is identified by a low-order model, whose transfer function and state-space equation are:

(1) $$G_{n}\left(s\right)=\frac{a_{n_{s}-1}s^{n_{s}-1}+\cdots +a_{1}s+a_{0}}{s^{n_{s}}+b_{n_{s}-1}s^{n_{s}-1}+\cdots +b_{1}s+b_{0}}\;\left[\frac{\mu m}{V}\right]$$

(2) $$\left\{\begin{array}{c} \dot{x}=\mathrm{Ax}+Bu\\ y=\mathrm{Cx} \end{array}\right.$$

where $n_{s}$ is the system order ($2⩽n_{s}⩽3$); $a_{i}$ ($i=0,1,\cdots ,n_{s}-1$) and $b_{j}$ ($j=0,1,\cdots ,n_{s}-1$) are constant coefficients. x is the continuous-time state vector; A, B, and C are continuous-time state-space matrices. Moreover, $G_{n}\left(s\right)$ is validated over $\left[\omega_{1},\omega_{2}\right]=2\pi \left[1,10000\right]$ rad/s.

Then, a continuous-time low-order dual-loop control scheme is developed based on the identified model. The Luenberger observer-based state feedback controller and tracking controller are, respectively, expressed as follows

(3) $$\left\{\begin{array}{c} \hat{x}=\left(A-\mathrm{LC}\right)\hat{x}+Bu+Ly\\ u_{s}=-K\hat{x}\\ u=u_{s}+u_{c} \end{array}\right.$$

(4) $$\left\{\begin{array}{c} u_{c}=C_{t}\left(s\right)e\\ C_{t}\left(s\right)=\frac{K_{I}}{s} \end{array}\right.$$

where $\hat{x}$ denotes the estimated continuous-time state vector, L is the observer matrix, K is the feedback gain matrix, and $K_{I}$ is the integral gain. According to the separation principle, the observer can be designed independently of the state feedback controller. With the assigned observer poles $\left(pl_{1},pl_{2},\cdots ,pl_{n_{s}}\right)$ and desired characteristic equation $\Phi \left(\lambda \right)=\left(\lambda -pl_{1}\right)\left(\lambda -pl_{2}\right)\cdots \left(\lambda -pl_{n_{s}}\right)$, the observer gain matrix L can be calculated via the Ackermann’s formula [30], namely $L=\Phi \left(A\right)P_{o}^{-1}{\left[0,\cdots ,0,1\right]}^{T}$, where $P_{o}={\left[C,\mathrm{CA},\cdots ,{\mathrm{CA}}^{n_{s}-1}\right]}^{T}$ is the observability matrix. Furthermore, in order to tune parameters K and $K_{I}$ to make the closed-loop magnitude response approximate the 0 dB line within the specified frequency range, the optimization objective function is defined as

(5) $$\begin{array}{c} \arg\min\left(\max_{\omega \in \left[\omega_{1},\omega_{2}\right]}20\log_{10}\left({\left|G_{cn}\left(s\right)\right|}_{s=j\omega }\right)\right)\\ s.t.M_{min}⩽M⩽M_{max} \end{array}$$

where $G_{cn}\left(s\right)=\frac{C{\left[sI-\left(A-\mathrm{BK}\right)\right]}^{-1}BC_{t}\left(s\right)}{1+C{\left[sI-\left(A-\mathrm{BK}\right)\right]}^{-1}BC_{t}\left(s\right)}$ is the the continuous-time closed-loop transfer function. ${\left.M\right|}_{n_{s}=2}=\left[\xi_{d},\omega_{d},K_{I}\right]$ or ${\left.M\right|}_{n_{s}=3}=\left[p_{d1},\xi_{d},\omega_{d},K_{I}\right]$ is the parameter vector to be optimized, which is constrained by $M_{min}$ and $M_{max}$. And K is calculated by ${\left.\det\left(\lambda I-\left(A-\mathrm{BK}\right)\right)\right|}_{n_{s}=2}=\left(\lambda -\xi_{d}\omega_{d}-\omega_{d}\sqrt{1-\xi_{d}^{2}}i\right)\left(\lambda -\xi_{d}\omega_{d}+\omega_{d}\sqrt{1-\xi_{d}^{2}}i\right)$ or ${\left.\det\left(\lambda I-\left(A-\mathrm{BK}\right)\right)\right|}_{n_{s}=3}=\left(\lambda -p_{d1}\right)\left(\lambda -\xi_{d}\omega_{d}-\omega_{d}\sqrt{1-\xi_{d}^{2}}i\right)\left(\lambda -\xi_{d}\omega_{d}+\omega_{d}\sqrt{1-\xi_{d}^{2}}i\right)$. The optimal $M_{o}$ is obtained via the differential evolution algorithm [17].

Finally, to carry out experiments in the actual digital control system, the continuous-time controller is discretized to obtain a discrete-time controller via zero-order holder (ZOH) method, whose expression are

(6) $$\left\{\begin{array}{c} u_{c}^{d}\left(k\right)=C_{t}^{d}\left(z\right)e^{d}\left(k\right)\\ u_{s}^{d}\left(k\right)=-K{\hat{x}}^{d}\left(k\right)\\ u^{d}\left(k\right)=u_{s}^{d}\left(k\right)+u_{c}^{d}\left(k\right)\\ {\hat{x}}^{d}\left(k+1\right)={\left(A-\mathrm{LC}\right)}^{d}\hat{x}\left(k\right)+B^{d}u^{d}\left(k\right)+L^{d}y^{d}\left(k\right) \end{array}\right.$$

where $C_{t}^{d}$ is the discretized integral controller, ${\hat{x}}^{d}\left(k\right)$ is the estimated discrete state at the *k*-th instant, and $B^{d}$, $L^{d}$ and ${\left(A-\mathrm{LC}\right)}^{d}$ are the discretized state-space matrices.

### 2.2. Problem Description

As described above, in the conventional dual-loop control framework, the theoretical magnitude response $G_{cn}\left(s\right)$ is derived under the condition $x\left(t\right)=\hat{x}\left(t\right)$. However, the continuous-time observer and integrator must be discretized and then be applied in the digital control system. Due to magnitude and phase errors introduced by the ZOH discretization [24,25,26], the estimated discrete-time state vector ${\hat{x}}^{d}\left(k\right)$ is not equal to the actual state vector ${\left.x\left(t\right)\right|}_{t=kT_{s}}$. Moreover, actual integral control vulue $u_{c}^{d}\left(k\right)=C_{t}^{d}\left(z\right)e^{d}\left(k\right)$ is also not equal to the theoretical value ${\left.u_{c}\left(t\right)\right|}_{t=kT_{s}}$. Ultimately, the amplitude spectrum obtained in the experiment diverges from its theoretical prediction. Furthermore, when the model mismatch exists between the nominal model and the actual system, the actual control performance degradation will become worse. In addition, although the differential evolution algorithm can overcome the system order limitation, they are computationally time-consuming and prone to local optima, especially for parameter tuning of high-order controllers.

The problem addressed in this study is to develop a dual-loop control framework based on the discrete-time domain and high-order model, ensuring the consistency between the achieved bandwidth and theoretical bandwidth. In this study, the developed control framework is designed directly in the discrete-time domain without the need for discretization. Moreover, the DLQR technique is utilized to quickly tune controller parameters and achieve a wider theoretical amplitude spectrum.

## 3. Developed Dual-Loop Control Framework

**Figure 2:**
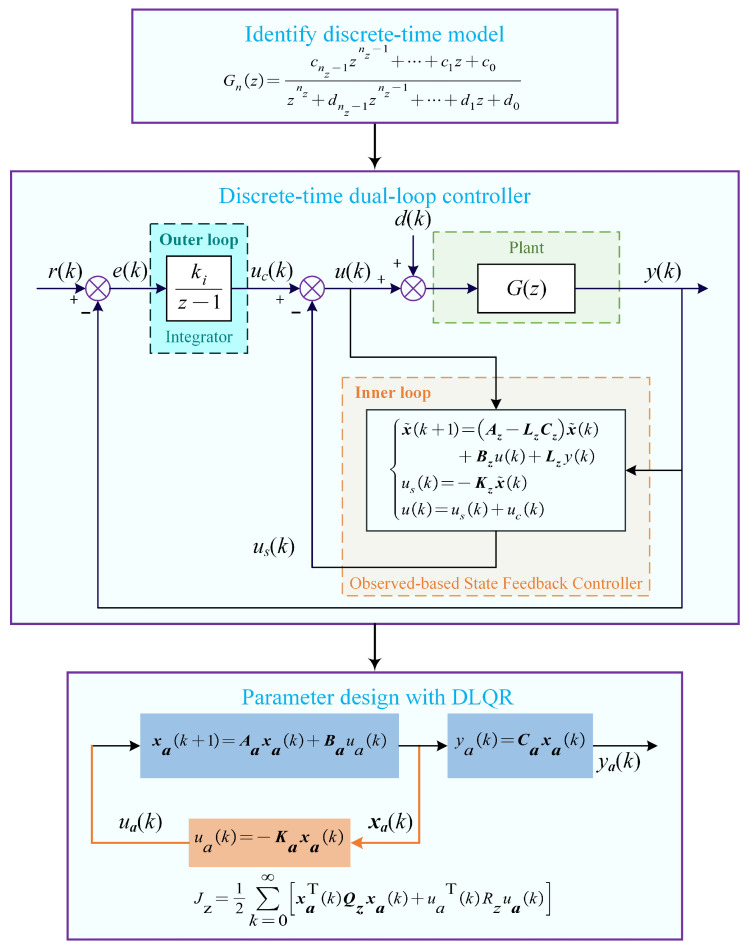
Design procedure of the developed discret-time high-order dual-loop controller framework.

Figure 2 presents the design procedure for the developed dual-loop control framework. Next, we will elaborate on the controller’s structure, parameter design, and stability analysis.

### 3.1. Controller Structure

The open-loop system dynamics are accurately described through a discrete-time high-order model, whose pulse transfer function and state-space representations are given as follows:

(7) $$G\left(z\right)=\frac{c_{n_{z}-1}z^{n_{z}-1}+\cdots +c_{1}z+c_{0}}{z^{n_{z}}+d_{n_{z}-1}z^{n_{z}-1}+\cdots +d_{1}z+d_{0}}$$

(8) $$\left\{\begin{array}{c} x\left(k+1\right)=A_{z}x\left(k\right)+B_{z}u\left(k\right)\\ y\left(k\right)=C_{z}x\left(k\right) \end{array}\right.$$

where $n_{z}$ is the discrete-time system order ($n_{z}⩾4$); $c_{i}$ ($i=0,1,\cdots ,n_{z}-1$) and $d_{j}$ ($j=0,1,\cdots ,n_{z}-1$) are constant coefficients. u(k), y(k) and x(k) are input, output, and state vectors of the system at *k*-th instant, respectively; $A_{z}$, $B_{z}$ and $C_{z}$ are discrete-time state space matrices, which is derived from G(z).

As depicted in Figure 2, in the outer loop, a discrete-time integrator $C_{t}\left(z\right)=\frac{k_{i}}{z-1}$ obtained via the ZOH discretization is utilized to eliminate steady-state errors, where $k_{i}$ is the discrete-time integral gain. The outer-loop control input is calculated by $u_{c}\left(k\right)=C_{t}\left(z\right)e\left(k\right)$. In the inner-loop, the discrete-time Luenberger observer-based state feedback controller is designed to enhance system damping according to the identified discrete-time high-order model. The discrete-time observer and state feedback controller are expressed as follows

(9) $$\left\{\begin{array}{c} \tilde{x}\left(k+1\right)=\left(A_{z}-L_{z}C_{z}\right)\tilde{x}\left(k\right)+B_{z}u\left(k\right)+L_{z}y\left(k\right)\\ u_{s}\left(k\right)=-K_{z}\tilde{x}\left(k\right) \end{array}\right.$$

where $\tilde{x}\left(k\right)$ is the estimated discrete-time state vector, $L_{z}$ is the discrete-time observer matrix, $K_{z}$ is the discrete-time state feedback gain matrix, and u(k) is the actual control input of the controlled system, calculated by $u\left(k\right)=u_{c}\left(k\right)-u_{s}\left(k\right)$.

### 3.2. Parameter Design

Analogous to the continuous-time dual-loop control framework, the observer and controller parameters in the discrete-time dual-loop framework can be designed decoupled. Additionally, to achieve wide closed-loop bandwidth, $k_{i}$ and $K_{z}$ must be simultaneously tuned.

For the discrete-time observer, $L_{z}$ is computed via the discrete-time Ackermann’s formula [31], given by $L=\Phi_{z}\left(A_{z}\right)P_{oz}^{-1}{\left[0,\cdots 0,1\right]}^{T}$, where $pz_{1},pz_{2},\cdots ,pz_{n_{z}}$ are desired observer poles in the *z*-plane, $P_{oz}={\left[C_{z},C_{z}A_{z},\cdots ,C_{z}A_{z}^{n_{z}-1}\right]}^{T}$ is the discrete-time observability matrix, and $\Phi_{z}\left(\lambda_{z}\right)=\left(\lambda_{z}-pz_{1}\right)\left(\lambda_{z}-pz_{2}\right)\cdots \left(\lambda_{z}-pz_{n_{z}}\right)$ is the desired discrete-time characteristic equation.

For discrete-time controllers, we first incorporate an additional integral substate $x_{I}\left(k\right)$, governed by $x_{I}\left(k+1\right)=x_{I}\left(k\right)+e\left(k\right)$. Since $e\left(k\right)=r\left(k\right)-y\left(k\right)=r\left(k\right)-C_{z}x\left(k\right)$, it yields $x_{I}\left(k+1\right)=x_{I}\left(k\right)+r\left(k\right)-C_{z}x\left(k\right)$. Then, by augmenting the original system state x(k) with $x_{I}\left(k\right)$, the new state of the augmented system becomes $x_{a}\left(k\right)={\left[x\left(k\right),x_{I}\left(k\right)\right]}^{T}$. Meanwhile, the state feedback control law of the augmented system becomes $u_{a}\left(k\right)=u_{s}\left(k\right)+u_{c}\left(k\right)=C_{t}\left(z\right)e\left(k\right)-K_{z}x\left(k\right)=k_{i}x_{I}\left(k\right)-K_{z}x\left(k\right)$. Moreover, the output of the augmented system $y_{a}\left(k\right)$ is still y(k). Finally, the discrete-time state space equation and state feedback control law of the augmented system are, respectively, expressed as.

(10) $$\begin{array}{c} \left\{\begin{array}{c} x_{a}\left(k+1\right)=A_{a}x_{a}\left(k\right)+B_{a}u_{a}\left(k\right)+N_{a}r\left(k\right)\\ y_{a}\left(k\right)=C_{a}x_{a}\left(k\right) \end{array}\right. \end{array}$$

(11) $$u_{a}\left(k\right)=-K_{a}x_{a}\left(k\right)$$

where $A_{a}=\left[\begin{array}{cc} A_{z} & 0_{n_{z}\times 1}\\ -C_{z} & 1 \end{array}\right]$, $B_{a}=\left[\begin{array}{c} B_{z}\\ 0 \end{array}\right]$, $N_{a}=\left[\begin{array}{c} 0_{n_{z}\times 1}\\ 1 \end{array}\right]$, $C_{a}=\left[\begin{array}{cc} C_{z} & 0 \end{array}\right]$, and $K_{a}=\left[\begin{array}{cc} K_{z} & -k_{i} \end{array}\right]$. Obviously, the parameter optimization problem of the original system is transformed into the state regulation problem for the augmented system. Therefore, the simple and efficient DLQR technique [32] can be used. Specifically, through selecting a diagonally symmetric positive definite state weighting matrix $Q_{z}$ and a positive scalar $R_{z}$, the feedback gain matrix $K_{a}$ can be obtained by minimizing the following cost function:

(12) $$J_{z}=\frac{1}{2}\sum_{k=0}^{\infty }\left[x_{a}^{T}\left(k\right)Q_{z}x_{a}\left(k\right)+u_{a}^{T}\left(k\right)R_{z}u_{a}\left(k\right)\right]$$

where $Q_{z}=\mathrm{diag}\left(qx_{1},\cdots ,qx_{nz},qi\right)$ includes normal substate weights $qx_{i}\left(i=1,\cdots ,n_{z}\right)$ and integral substate qi.

**Remark 1.**  *To maximize closed-loop bandwidth of the original system while preserving stability, the rule helping in picking the weighting matrice $Q_{z}$ and the scalar $R_{z}$ for DLQR are that (1) large weight is assigned to dominant integral substate qi to extend the original closed-loop bandwidth, (2) consistent weightings are assigned to non-dominant normal substates $qx_{i}$, simplifying the weights tuning process, and (3) the ratios $qi/qx_{i}$ and $qi/R_{z}$ are tuned to achieve a flat magnitude spectrum below 0 dB and ensure adequate stability margins in the original closed-loop system*.

### 3.3. Stability Analysis

According to the above analysis, the closed-loop stability of the original discrete-time dual-loop control system is equivalent to the stability of the augmented system under the DLQR. Next, we will establish a Lyapunov-based stability analysis for this controlled system.

Condisdering the discrte-time system $x_{a}\left(k+1\right)=A_{a}x_{a}\left(k\right)+B_{a}u_{a}\left(k\right)$ with control law $u_{a}\left(k\right)=-K_{a}x_{a}\left(k\right)$ and performance functional $J_{z}$, the control gain $K_{a}$ is given by $K_{a}={\left(R_{z}+B_{a}^{T}P_{z}B_{a}\right)}^{-1}B_{a}^{T}P_{z}A_{a}$, where P is a positive definite real symmetric matrix, satisfying the following discrete-time Algebraic Riccati Equation [32]:

(13) $$P_{a}=A_{a}^{T}P_{a}A_{a}-A_{a}^{T}P_{a}B_{a}{\left(R_{z}+B_{a}^{T}P_{z}B_{a}\right)}^{-1}B_{a}^{T}P_{z}A_{a}+Q_{z}$$

Then, the closed-loop system dynamics becomes $x_{a}\left(k+1\right)=A_{\mathrm{ca}}x_{a}\left(k\right)$, where $A_{\mathrm{ca}}=A_{a}-B_{a}K_{a}$. Defining a positve definite Lyapunov function $V\left(x_{a}\left(k\right)\right)=x_{a}^{T}\left(k\right)P_{a}x_{a}\left(k\right)$, the forward difference is obtained as

(14) $$\begin{array}{cc} \Delta V\left(x_{a}\left(k\right)\right) & =V\left(x_{a}\left(k+1\right)\right)-V\left(x_{a}\left(k\right)\right)\\ & =x_{a}^{T}\left(k+1\right)P_{a}x_{a}\left(k+1\right)-x_{a}^{T}\left(k\right)P_{a}x_{a}\left(k\right)\\ & ={\left(A_{\mathrm{ca}}x_{a}\left(k\right)\right)}^{T}P_{a}A_{\mathrm{ca}}x_{a}\left(k\right)-x_{a}^{T}\left(k\right)P_{a}x_{a}\left(k\right)\\ & =x_{a}^{T}\left(k\right)\left(A_{\mathrm{ca}}^{T}P_{a}A_{\mathrm{ca}}-P_{a}\right)x_{a}\left(k\right)\\ & =x_{a}^{T}\left(k\right)\left(-Q_{z}-K_{a}^{T}R_{z}K_{a}\right)x_{a}\left(k\right) \end{array}$$

Since $Q_{z}$ and $K_{a}^{T}R_{z}K_{a}$ are both positive definite, $\Delta V\left(x_{a}\left(k\right)\right)$ is negative definite for $x_{a}\left(k\right)\neq 0_{\left(n_{z}+1\right)\times 1}$. Therefore, according to the Lyapunov stability theorem for the discrete-time system, the developed discrete-time dual-loop control system with the DLQR is globally asymptotically stable.

## 4. Experimental Validation

In this section, the developed dual-loop control framework based on the discrete-time high-order model (DDLC-DHOM) is validated on a one-degree-of-freedom (1-DOF) piezo-actuated nanopositioning system. To illustrate the superiority of the developed framework, the results are compared with the conventional dual-loop control framework based on the continuous-time high-order model (CDLC-DLOM), where continuous-time LQR replaces the differential evolution algorithm for computationally efficient parameter tuning. In addition, in order to verify the performance degradation caused by the model mismatch, the results are also compared with the dual-loop control framework based on the discrete-time low-order model (DLC-DLOM).

### 4.1. Experimental Setup

**Figure 3:**
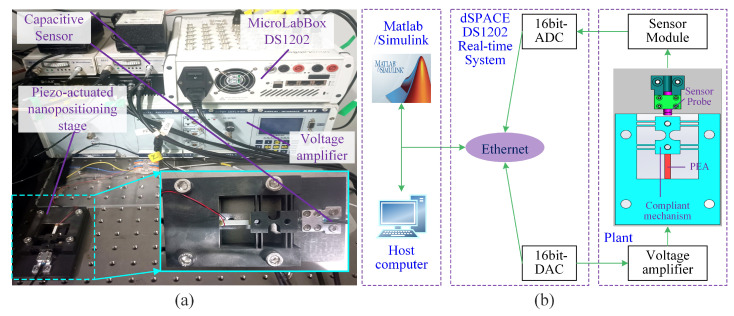
Experimental setup: (**a**) the experimental system and (**b**) the block diagram of system.

As shown in Figure 3, the experimental system consists of a 1-DOF piezo-actuated nanopositioning stage (compliant mechanism: Steel-45; PEA: PSt150−3.5×3.5×20, Tokin, Shiroishi, Japan), a voltage amplifier (E00-D3, Coremorrow, Harbin, China), a real-time simulation system (MicroLabBox-DS1202, dSPACE, Paderborn, Germany), a capacitive displacement sensor (8810-2823, MicroSense, CA, USA), and a host computer. The control system model is first built in Matlab∖Simulink environment. Then, the Simulink model is compiled into code. Finally, the code runs on the dSPACE Base Board for real-time implementation of the controller. During the control system execution, the control voltage is fed into the amplifier through a 16-bit digital-to-analog converter (DAC), and the output displacement of the stage measured by the capacitive displacement sensor is fed back to the controller through a 16-bit analog-to-digital converter (ADC).

Typically, a sufficiently high sampling frequency $f_{s}$ is desired to achieve closed-loop control for high-speed signals. However, in dSPACE hardware-in-the-loop experiments, the digital controller’s single-cycle execution involves ADC and DAC conversions, control algorithm computation and execution, data register and access operations. To ensure the real-time implementation of the controller, the sample frequency must satisfy $f_{s}<1/\tau_{e}$, where $\tau_{e}$ denotes the actual controller execution time. Through multiple experimental measurements, the sample frequency $f_{s}$ is set to be 50 kHz (i.e., the sampling period $T_{s}=0.00002$ s) to avoid program overrun.

### 4.2. System Identification

To avoid the coupling effect of the hysteresis, the creep, and the linear dynamics, a fast square wave signal with the period of 10 ms is excited to the system [23]. By using the Matlab 2020a system identification toolbox, the discrete-time high-order model of the open-loop system for DDLC-DHOM is identified from the input–output data with the *armax* algorithm as

(15) $$G_{n}\left(z\right)=\frac{c_{4}z^{4}+c_{3}z^{3}+c_{2}z^{2}+c_{1}z+c_{0}}{z^{5}+d_{4}z^{4}+d_{3}z^{3}+d_{2}z^{2}+d_{1}z+d_{0}}$$

where the fit to estimation data is 98.64 %. Table 1 gives all parameters of the identified model $G_{n}\left(z\right)$. By disregarding the system’s high-order dynamics, the identified discrete-time low-order model for DLC-DLOM is obtained as

(16) $$G_{l}\left(z\right)=\frac{0.169z^{2}-0.4666z+0.4129}{z^{3}-2.155z^{2}+2.03z-0.7625}$$

Moreover, according to $G_{n}\left(z\right)$, the continuous-time high-order model for CDLC-CHOM is obtained using using *d2c* operation with ZOH as follows:

(17) $$G_{h}\left(s\right)=\frac{a_{h4}s^{4}+a_{h3}s^{3}+a_{h2}s^{2}+a_{h1}s+a_{h0}}{s^{5}+b_{h4}s^{4}+b_{h3}s^{3}+b_{h2}s^{2}+b_{h1}s+b_{h0}}$$

Table 2 gives all parameters of the transfer function $G_{h}\left(s\right)$.

**Figure 4:**
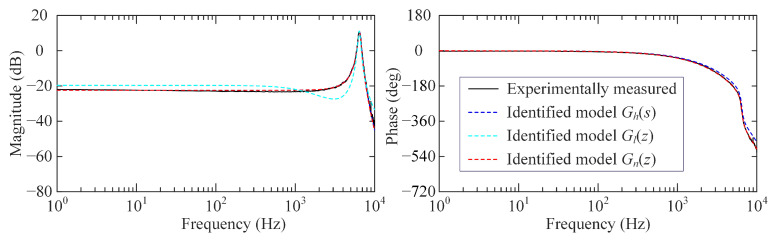
Comparison of frequency response by the experimentally measured and identified models.

Figure 4 shows the frequency response comparison results of experimentally measured and identified models, including $G_{l}\left(z\right)$, $G_{h}\left(s\right)$, and $G_{n}\left(z\right)$. Obviously, in contrast to the low-order model $G_{l}\left(z\right)$, the high-order model $G_{n}\left(z\right)$ and $G_{h}\left(s\right)$ both exhibit superior capability in capturing the actual system dynamics within a wider frequency range. Remarkably, due to the phase lag introduced by the zero-order hold, $G_{n}\left(z\right)$ exhibits a $\omega T_{s}/2$ phase lag compared with $G_{h}\left(s\right)$.

By solving the polynomial pole placement equations of $G_{l}\left(z\right)$, $G_{h}\left(s\right)$ and $G_{n}\left(z\right)$, it can be found that they are all stable. Additionally, it can be verified that their controllability and observability matrices are all full rank. Therefore, their poles can be arbitrarily assigned through state feedback controllers. Subsequently, above identified system models will, respectively, be used for controller implementation and experimental validation according to Section 2.1 and Section 3.2.

### 4.3. Experimental Results

For DDLC-DHOM, we set $Q_{\mathrm{hz}}=\left[\begin{array}{c} 5\times 10^{4},0,0,0,0,0\\ 0,5\times 10^{4},0,0,0,0\\ 0,0,5\times 10^{4},0,0,0\\ 0,0,0,5\times 10^{4},0,0\\ 0,0,0,0,5\times 10^{4},0\\ 0,0,0,0,0,8\times 10^{6} \end{array}\right]$ and $R_{\mathrm{hz}}=10^{6}$ with $G_{n}\left(z\right)$. Using *dlqr* operation of Matlab, the control parameters are calculated as $k_{i}=1.2073$ and $K_{z}=\left[2.7906,-1.712,1.5069,-0.5965,0.7956\right]$, respectively.

For DLC-DLOM, we set $Q_{\mathrm{lz}}=\left[\begin{array}{c} 1\times 10^{3},0,0,0\\ 0,1\times 10^{3},0,0\\ 0,0,1\times 10^{3},0\\ 0,0,0,1.4794\times 10^{5} \end{array}\right]$ and $R_{\mathrm{lz}}=10^{5}$ with $G_{l}\left(z\right)$, The *dlqr* operation is also used to simultaneously tuning controller parameters, resulting in $K_{\mathrm{lz}}=\left[0.8435,-1.1074,0.7894\right]$ and $k_{li}=0.7296$.

Due to the high-order characteristics of $G_{h}\left(s\right)$, it is hard for intelligent optimization algorithms to optimize the system bandwidth and customize the desired bandwidth. Therefore, for CDLC-CHOM, the continuous-time LQR technique is employed for tuning controller parameters. We set $Q_{\mathrm{hs}}=\left[\begin{array}{c} 1\times 10^{2},0,0,0,0,0\\ 0,1\times 10^{2},0,0,0,0\\ 0,0,1\times 10^{2},0,0,0\\ 0,0,0,1\times 10^{2},0,0\\ 0,0,0,0,1\times 10^{2},0\\ 0,0,0,0,0,6.512\times 10^{8} \end{array}\right]$ and $R_{\mathrm{hs}}=10^{-1}$. Using *lqr* operation in the Matlab, the integral control gain and the state feedback matrix are obtained as $K_{\mathrm{hs}}=\left[242.9410,497.7668,693.5323,315.9430,547.3478\right]$ and $K_{hI}=80696.964$, respectively.

**Figure 5:**
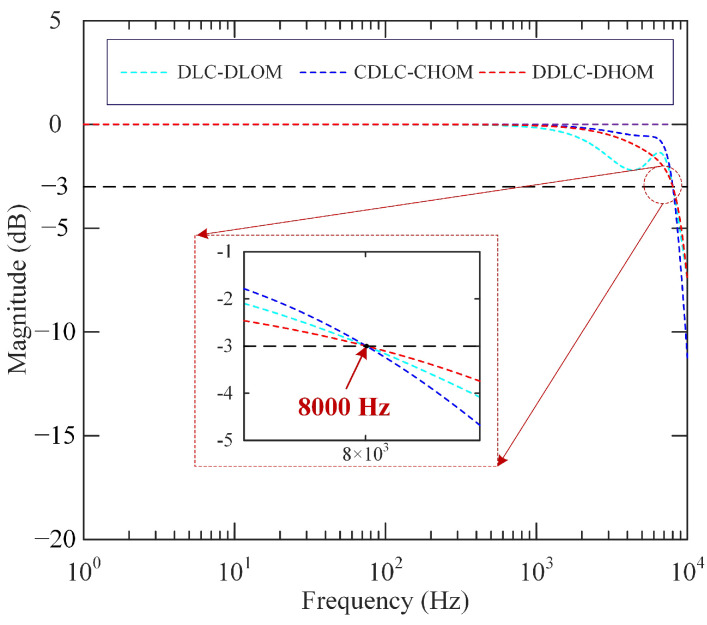
Theoretical magnitude responses of the closed-loop systems.

According to these control parameters, theoretical closed-loop magnitude responses of three controlled systems are depicted in Figure 5. It is observed that the closed-loop system bandwidth is set to be about 8000 Hz, which is greater than the natural frequency of the open-loop system. Moreover, the amplitude and phase margins of the above three controlled systems can be calculated using MATLAB. They are 6.37 dB/60.6°(DDLC-DHOM), 6.92 dB/61.4°(DLC-DLOM) and 6.27dB/60.6°(CDLC-CHOM), respectively. It shows the controlled systems have close and sufficient stability margins.

For fairly comparing DLC-DLOM, CDLC-CHOM, and DDLC-DHOM, the desired observer bandwidths are set to be the same for the three controlled systems, namely $\omega_{o}=2\pi \cdot 9000$ rad/s. Based on the bandwidth parameterization method [33] and using *acker* operation, the observer gain matrices are, respectively, calculated as $L_{\mathrm{lz}}={\left[-3.1428,-0.0914,4.0559\right]}^{T}$, $L={\left[148.5892,1429.7,-955.588,-3069.9,1343.9\right]}^{T}$, and $L_{z}={\left[\begin{array}{c} -2.7207,-5.9985,-2.6076,1.4376,1.7174 \end{array}\right]}^{T}$.

To comprehensively evaluate the control performance of the three frameworks, step response, trajectory tracking, and frequency response tasks are designed and applied to the piezo-actuated nanopositioning system.

**Figure 6:**
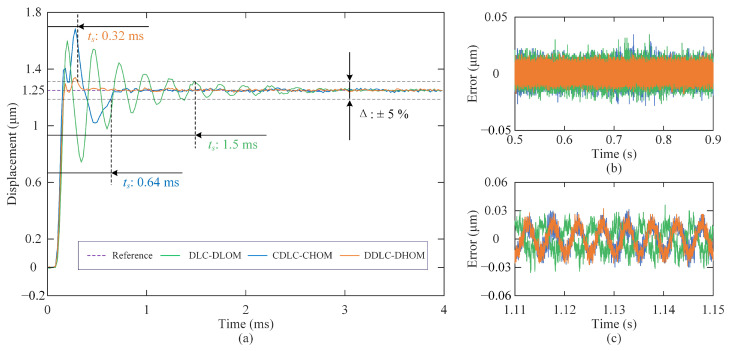
Step responses of the closed-loop systems: (**a**) 0–4 ms; (**b**) 0.5–0.9 s; (**c**) 1.11–1.15 s.

**(1) Step Response:** The step responses of closed-loop systems obtained with DLC-DLOM, CDLC-CHOM, and DDLC-DHOM are plotted in Figure 6a, where the settling time of the step response is defined with a 5% error band. It is observed that the system under DDLC-DHOM demonstrates optimal dynamic performance, with a settling time of only 0.32 ms. Compared to CDLC-CHOM and DLC-DLOM, the response speed of DDLC-DHOM is improved by 50% and 78.7%, respectively. Additionally, systems under CDLC-CHOM and DLC-DLOM exhibit significant overshoot, leading to system instability and safety risks. In contrast, the system under DDLC-DHOM demonstrates minimal overshoot, resulting in negligible impact on practical applications. Figure 6b displays the steady-state error curve of the step response, where the root mean square of noise for controlled systems with DLC-DLOM, CDLC-CHOM, and DDLC-DHOM are calculated as 7.0 nm, 7.4 nm, and 5.5 nm, respectively. This means that the system under DDLC-DHOM has better noise suppression capability. Furthermore, a sinusoidal wave input disturbance $d=0.1\sin\left(2\pi \cdot 200\right)V$ is injected at t=1.1 s during the step response test. As illustrated in Figure 6c, the root mean square of the steady-state error for controlled systems with DLC-DLOM, CDLC-CHOM, and DDLC-DHOM are calculated as 12.5 nm, 12.7 nm, and 12.2 nm, respectively. This reveals comparable disturbance rejection capabilities among the three control frameworks.

**(2) Trajectory Tracking:** The sinusoidal wave (SW) and triangular wave (TW) are widely used in nanopositioning applications. Therefore, SW and TW signals with the fundamental frequencies of 10, 50, 200, 500, 1000, and 1250 Hz are tested. It should be noted that for nanopositioning applications, such as the AFM scanning and imaging, perfectly delayed tracking is better than imperfect timely tracking [17,29], the shifted output is utilized to quantify tracking results via the time-delay post-processing. Hence, the root mean square error $e_{\mathrm{rms}}$ and the maximum absolute error $e_{\max}$ are selected as an indicator to assess the tracking performance, which are redefined as

(18) $$e_{\mathrm{rms}}=\sqrt{\frac{1}{N}\sum_{k=1}^{N}{\left(r\left(k\right)-y\left(k-n_{d}\right)\right)}^{2}}\;\;\left(\mathrm{nm}\right)$$

(19) $$e_{\max}=\max\left|r\left(k\right)-y\left(k-n_{d}\right)\right|\;\;\left(\mathrm{nm}\right)$$

where *N* is the total number of sampling data. $n_{d}$ is the optimal time delay term, determined by $n_{d}=\underset{n}{\arg\min}\;\;\left[\max_{k\in \left[1,N\right]}\left|r\left(k\right)-y\left(k-n\right)\right|\right]$.

**Figure 7:**
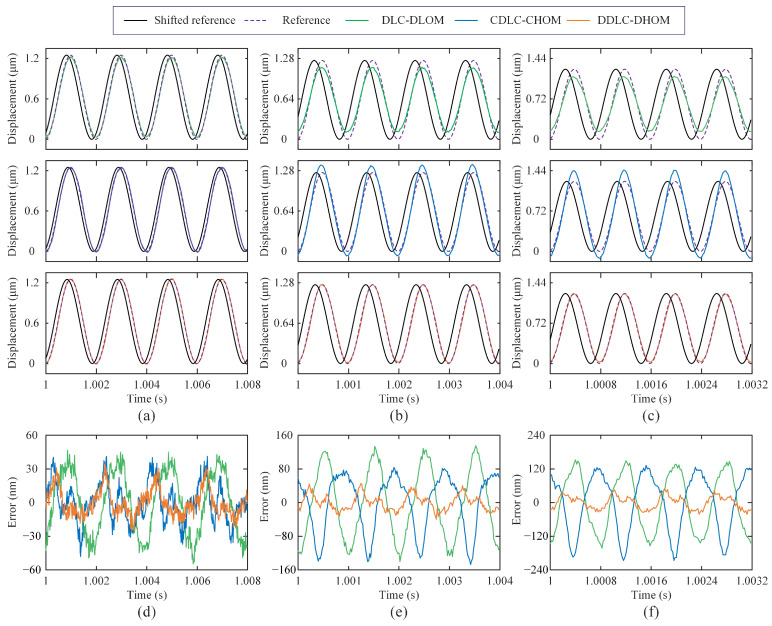
Experimental results of tracking SWs with three control frameworks: (**a**) tracking results—500 Hz; (**b**) tracking results—1000 Hz; (**c**) tracking results—1250 Hz; (**d**) tracking errors—500 Hz; (**e**) tracking errors—1000 Hz; (**f**) tracking errors—1250 Hz.

**Figure 8:**
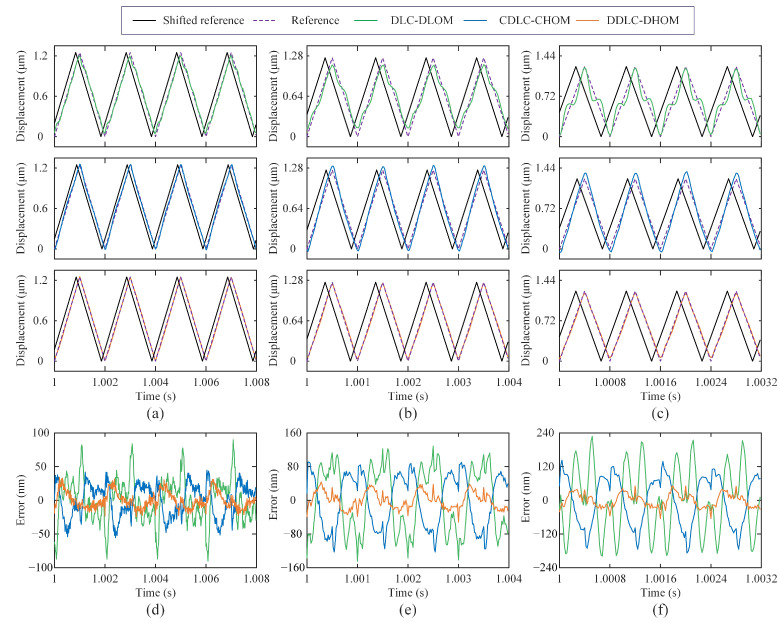
Experimental results of tracking TWs with three control frameworks: (**a**) tracking results—500 Hz; (**b**) tracking results—1000 Hz; (**c**) tracking results—1250 Hz; (**d**) tracking errors—500 Hz; (**e**) tracking errors—1000 Hz; (**f)** tracking errors—1250 Hz.

The statistical results of tracking errors for three control frameworks are summarized in Table 3, where the mean and standard deviation of $e_{\mathrm{rms}}$ and $e_{\max}$ are based on 10 repeated runs. Meanwhile, to visually compare the tracking results, time-domain experimental results for SW and TW signals with 200 Hz, 1000 Hz, and 1250 Hz from a single run are plotted in Figure 7 and Figure 8, respectively. Obviously, subfigures (a–c) in Figure 7 and Figure 8 show that the delay alignment between the output and the reference significantly improved the tracking results. According to statistical results, it can be seen that three control frameworks exhibit comparable tracking errors in low-frequency (⩽200 Hz) trajectory tracking. For high-frequency (>200 Hz) trajectories tracking, DDLC-DHOM demonstrates the most superior tracking performance. Particularly, the accuracy ($e_{\mathrm{rms}}$/$e_{\max}$) of DDLC-DHOM has improved by 78.1%/71.5% and 71.7%/63.4% for the 1250 Hz TW signal, respectively, compared with that of DLC-DLOM and CDLC-CHOM, which demonstrates its excellent performance in the high-speed scanning motion.

**Figure 9:**
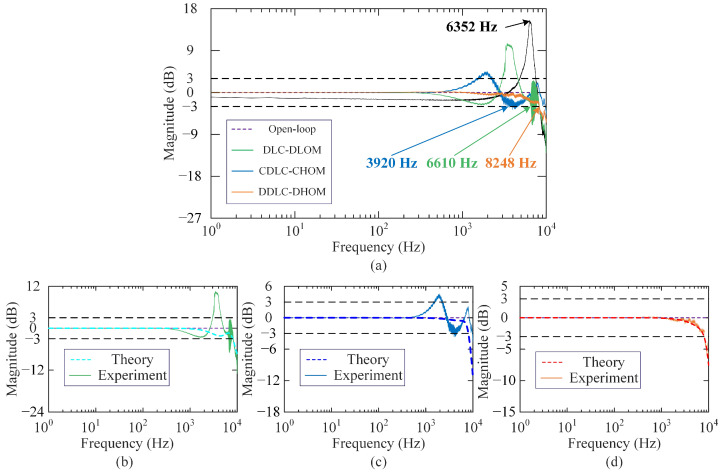
Amplitude spectrum results: (**a**) experimental amplitude spectrums of the open loop system and closed-loop systems with DLC-DLOM, CDLC-CHOM, and DDLC-DHOM; comparative result of experimental and theoretical amplitude spectrum for (**b**) DLC-DLOM; (**c**) CDLC-CHOM; (**d**) DDLC-DHOM.

**(3) Frequency response:** Through swept-sine excitation, experimental amplitude spectrums are obtained and shown in Figure 9a. From Figure 9a, the experimental bandwidth of the open-loop system is 6352 Hz, and the experimental bandwidths of the closed-loop system by DLC-DLOM, CDLC-CHOM, and DDLC-DHOM frameworks are measured as 3920 Hz, 6610 Hz, and 8248 Hz, respectively. Obviously, only the actual bandwidth of the system with DDLC-DHOM is larger than the first-order resonant frequency of the open-loop system and approximates the theoretical design bandwidth 8000 Hz. Furthermore, the comparative results of experimental and theoretical amplitude spectra for three frameworks are illustrated in Figure 9b, Figure 9c, and Figure 9d, respectively. The actual amplitude spectrum of the systems with DDLC-DHOM agrees well with the theoretical ones, while the systems controlled by DLC-DLOM and CDLC-CHOM exhibit a significant discrepancy between practical and theoretical magnitude responses. This means that performance degradation caused by lower-order modeling errors and controller discretization cannot be neglected for high-bandwidth control requirements. In addition, the developed framework based on a discrete-time high-order model can achieve the goal of high-bandwidth precision control by avoiding modeling and discretization errors as well as completely hysteresis suppression.

## 5. Conclusions

In this paper, a discrete-time high-order dual-loop control framework is developed to improve the control bandwidth of a nanopositioning system with high-order dynamics and hysteresis nonlinearity, where the DLQR technique is used to tune control parameters. The DDLC-DHOM framework is directly synthesized in the discrete-time domain, bypassing the need for conventional continuous-controller discretization, and designed through a high-order model, eliminating modeling and controller errors. Comparative experiments are conducted on a 1-DOF piezo-actuated nanopositioning system. Results show that compared to DLC-DLOM and CDLC-CHOM, the developed framework achieves bandwidth improvements of 4328 Hz and 1368 Hz, respectively, and exceeds the open-loop first-order resonant frequency by 1896 Hz. More importantly, its experimental magnitude response closely matches the theoretical design, indicating complete hysteresis suppression. Additionally, it is also confirmed that the DDLC-DHOM frame exhibits the fastest dynamic response and produces the smallest tracking errors of sinusoidal and triangular signals. These suggest that the developed framework is highly effective for high-bandwidth control and offers distinct advantages in nanopositioning applications requiring rapid scanning and positioning.

Future research focuses on extending the proposed framework to more complicated systems, such as multi-input multi-output (MIMO) systems and multi-resonance systems.

## Figures and Tables

**Table 1:** Numerical coefficients of the model $G_{h}\left(z\right)$.

$c_{4}$	$c_{3}$	$c_{2}$	$c_{1}$	$c_{0}$
−0.003362	0.01011	−0.01613	0.06889	0.09101
$d_{4}$	$d_{3}$	$d_{2}$	$d_{1}$	$d_{0}$
−2.437	2.918	−1.833	0.6346	−0.1089

**Table 2:** Numerical coefficients of the model $G_{h}\left(s\right)$.

$a_{h4}$	$a_{h3}$	$a_{h2}$	$a_{h1}$	$a_{h0}$
5523	$-8.55\times 10^{8}$	$9.233\times 10^{13}$	$-5.529\times 10^{18}$	$1.514\times 10^{23}$
$b_{h4}$	$b_{h3}$	$b_{h2}$	$b_{h1}$	$b_{h0}$
$1.109\times 10^{5}$	$7.993\times 10^{9}$	$3.048\times 10^{14}$	$1.023\times 10^{19}$	$1.743\times 10^{23}$

**Table 3:** Tracking performance under different control frameworks for different trajectories.

Trajectory	Frequency	Index	DLC-DLOM	CDLC-CHOM	DDLC-DHOM
SW	10	$e_{\mathrm{rms}}$/$e_{\max}$	6.88 ± 0.29/27.55 ± 2.79	7.53 ± 0.18/29.76 ± 2.32	5.47 ± 0.06/18.81 ± 1.35
	50	$e_{\mathrm{rms}}$/$e_{\max}$	7.15 ± 0.39/27.37 ± 5.46	7.49 ± 0.24/27.38 ± 4.50	5.46 ± 0.07/19.03 ± 1.38
	200	$e_{\mathrm{rms}}$/$e_{\max}$	8.08 ± 0.38/26.46 ± 3.26	9.42 ± 0.18/30.55 ± 1.19	7.23 ± 0.07/23.16 ± 1.81
	500	$e_{\mathrm{rms}}$/$e_{\max}$	27.91 ± 0.13/56.72 ± 2.05	18.11 ± 0.27/49.38 ± 3.59	11.80 ± 0.27/35.21 ± 2.65
	1000	$e_{\mathrm{rms}}$/$e_{\max}$	82.47 ± 0.14/138.65 ± 1.8	70.85 ± 0.46/155.88 ± 8.93	18.08 ± 0.26/50.76 ± 3.98
	1250	$e_{\mathrm{rms}}$/$e_{\max}$	100.27 ± 0.14/160.29 ± 2.62	7.6 ± 0.3/215.68 ± 3.49	22.98 ± 0.21/55.23 ± 6.77
TW	10	$e_{rms}$/$e_{\max}$	7.22 ± 0.29/28.61 ± 4.99	7.56 ± 0.32/29.25 ± 4.51	5.32 ± 0.08/18.81 ± 1.65
	50	$e_{\mathrm{rms}}$/$e_{\max}$	7.55 ± 0.45/29.56 ± 6.09	8.57 ± 0.32/30.36 ± 5.00	5.49 ± 0.07/17.98 ± 1.71
	200	$e_{\mathrm{rms}}$/$e_{\max}$	11.56 ± 0.39/53.24 ± 3.10	24.19 ± 0.29/58.01 ± 4.09	7.38 ± 0.09/25.21 ± 1.78
	500	$e_{\mathrm{rms}}$/$e_{\max}$	32.69 ± 0.26/98.60 ± 4.25	11.56 ± 0.39/53.24 ± 3.10	12.54 ± 0.13/36.25 ± 1.8
	1000	$e_{\mathrm{rms}}$/$e_{\max}$	77.33 ± 0.22/142.83 ± 4.23	63.99 ± 0.44/127.43 ± 2.77	19.65 ± 0.14/53.43 ± 0.79
	1250	$e_{\mathrm{rms}}$/$e_{\max}$	121.31 ± 0.54/231.87 ± 3.05	91.74 ± 0.35/200.01 ± 4.34	25.35 ± 0.14/77.70 ± 2.27

## Data Availability

The data that support the findings of this study are available from the corresponding author upon reasonable request.

## Abbreviations

The following abbreviations are used in this manuscript:

AFM	Atomic force microscope
PPF	Positive position feedback
PVPF	Positive velocity and position feedback
IRC	Integral resonant control
PAVPF	Positive acceleration, velocity, and position feedback
ZOH	Zero-order holder
DDLC-DHOM	Developed Dual-loop control framework based on the discrete-time high-order model
DLC-DLOM	Dual-loop control framework based on the discrete-time low-order model
CDLC-CHOM	Conventional Dual-loop control framework based on the continuous-time high-order model
DAC	Digital-to-analog
ADC	Analog-to-digital
SW	Sinusoidal wave
TW	Triangular wave
